# The application of a deep learning system developed to reduce the time for RT-PCR in COVID-19 detection

**DOI:** 10.1038/s41598-022-05069-2

**Published:** 2022-01-24

**Authors:** Yoonje Lee, Yu-Seop Kim, Da-in Lee, Seri Jeong, Gu-Hyun Kang, Yong Soo Jang, Wonhee Kim, Hyun Young Choi, Jae Guk Kim, Sang-hoon Choi

**Affiliations:** 1 Department of Emergency Medicine, College of Medicine, Hallym University, Chuncheon, South Korea; 2 Hallym Bioinformatics & Convergence Research Laboratory, Hallym Translation Research Center, Kangnam Sacred-Heart Hospital, Hallym University, Chuncheon, South Korea; 3 Department of Convergence Software, Hallym University, Chuncheon, South Korea; 4 Department of Laboratory Medicine, Kangnam Sacred Heart Hospital, Hallym University College of Medicine, 6, Singil-ro, 1-gil, Youngdeungpo-gu, Seoul, 07441 South Korea; 5 Department of Biomedical Engineering, Yonsei University, Wonju, South Korea

**Keywords:** Biotechnology, Computational biology and bioinformatics, Health care, Medical research

## Abstract

Reducing the time to diagnose COVID-19 helps to manage insufficient isolation-bed resources and adequately accommodate critically ill patients. There is currently no alternative method to real-time reverse transcriptase polymerase chain reaction (RT-PCR), which requires 40 cycles to diagnose COVID-19. We propose a deep learning (DL) model to improve the speed of COVID-19 RT-PCR diagnosis. We developed and tested a DL model using the long short-term memory method with a dataset of fluorescence values measured in each cycle of 5810 RT-PCR tests. Among the DL models developed here, the diagnostic performance of the 21st model showed an area under the receiver operating characteristic (AUROC), sensitivity, and specificity of 84.55%, 93.33%, and 75.72%, respectively. The diagnostic performance of the 24th model showed an AUROC, sensitivity, and specificity of 91.27%, 90.00%, and 92.54%, respectively.

## Introduction

Last year, 2020, was a time when all humanity grappled with new changes due to the coronavirus disease 2019 (COVID-19) pandemic. COVID-19 has presented a serious threat despite being caused by infectious agents that represent a miniscule mass even when combined. Even in this time of confusion, humanity has made every effort to survive, overcome the crisis and find order.

From a medical perspective, the COVID-19 pandemic has required us to be fast and accurate in four key activities: diagnosis, isolation, treatment, and tracking. Among these activities, rapid diagnosis is of paramount importance because the remaining 3 can proceed quickly and accurately only when preceded by a rapid diagnosis. Early diagnosis is essential because the response must be able to quickly block the spread of infection by rapidly applying the remaining 3 activities upon rapid diagnosis.

For the diagnosis of this infectious disease, the real-time reverse transcriptase polymerase chain reaction (RT-PCR) test is most widely used as a reference test. Unfortunately, despite the importance of rapid diagnosis, this test can take up to approximately 6 h from sampling and may require consecutive tests to discriminate false-negative and false-positive results^1^.

Various efforts have been made to quickly and accurately diagnose COVID-19 with the help of machine learning or artificial intelligence (AI) using information such as symptoms, chest X-ray (CXR) findings, computed tomography (CT) findings, and routine laboratory blood test results^2–7^. Clinically useful results have been reported; however, the corresponding methods cannot completely replace the RT-PCR test^2–7^. In addition, rapid diagnostic kits for detecting severe acute respiratory syndrome coronavirus 2 (SARS-CoV-2), such as loop-mediated isothermal amplification assays^8^ and immunoassays utilizing antigen–antibody responses^9,10^, have also been developed. However, their relative performances compared to those of conventional RT-PCR are limited, and the available infrastructure for sufficient application to suspected patients is not available.

Unlike the approaches of previous studies, we investigated whether the time taken for RT-PCR diagnosis can be reduced through a deep learning (DL) model developed in this study. Using raw data of fluorescence values in every cycle of RT-PCR, we developed and tested a DL model that can predict the results before completion of the RT-PCR test.

## Methods

### Study participants

We enrolled patients who visited a specialized outpatient department for COVID-19 triage or an emergency department to identify COVID-19 cases between 23 November 2020 and 19 January 2021. The raw data of RT-PCR curves determined to detect SARS-CoV-2 during this period were collected.

This study was approved by the Institutional Review Committee (HKS 2020-07-007) of Hallym University Kangnam Sacred Heart Hospital in Korea; consent was waived because the subjects' data were anonymized. This study was conducted in accordance with the STARD guidelines and regulations as a study related to the diagnostic accuracy of COVID-19 RT-PCR.

### Materials

RNA extraction was performed using the MagNa Pure 96 System (Roche Diagnostics, Rotkreuz, Switzerland). The reagent for the RT-PCR assay used in this study was the STANDARD M nCoV Real-Time Detection kit (SD Biosensor, Gyeonggi, South Korea), and a Bio-Rad CFX96 analyser was used (Bio-Rad Laboratories, Inc., Hercules, CA, USA).

### Data description

Raw data included RT-PCR information on specimens collected via nasopharyngeal swabs of patients who had undergone SARS-CoV-2 RT-PCR testing at Kangnam Sacred Heart Hospital. The raw data consisted of fluorescence values derived during the RT-PCR test of SARS-CoV-2, and the fluorescence values measured for a total of 40 cycles through the RT-PCR test were recorded for each sample from each patient.

Therefore, for each sample, 40 fluorescence values measured over 40 cycles were recorded, and a total of 5810 raw data in this form were collected. That is, in the raw data, fluorescence values were recorded in 40 columns for each sample over a total of 5810 rows.

### Development of the DL model

The RT-PCR results (positive or negative) were used as the output variable to train the models. A total of 40 models were developed and validated, from the model trained with the fluorescence value of the first RT-PCR cycle to the model trained from the fluorescence value of all 40 RT-PCR cycles.

For example, the first model was trained with the fluorescence value of the first RT-PCR cycle, and the second model was trained with the fluorescence values from the first to second RT-PCR cycles. In the same way, the 39th model was trained with the fluorescence values from the first to the 39th RT-PCR cycle, and the 40th model was trained with the fluorescence values from the first to the 40th RT-PCR cycle.

The raw RT-PCR test data were obtained from the first cycle to the 40th cycle according to the passage of time. In other words, the raw data were collected in a time series. The RT-PCR test is a diagnostic method based on the time when the fluorescence value reaches a threshold value by measuring the fluorescence value measured at each cycle.

Thus, for the model development in this study, we applied the long-term short memory (LSTM) method, which is typically used to address the vanishing gradient problem with existing recurrent neural networks (RNNs) for time series data.

**Figure 1:**
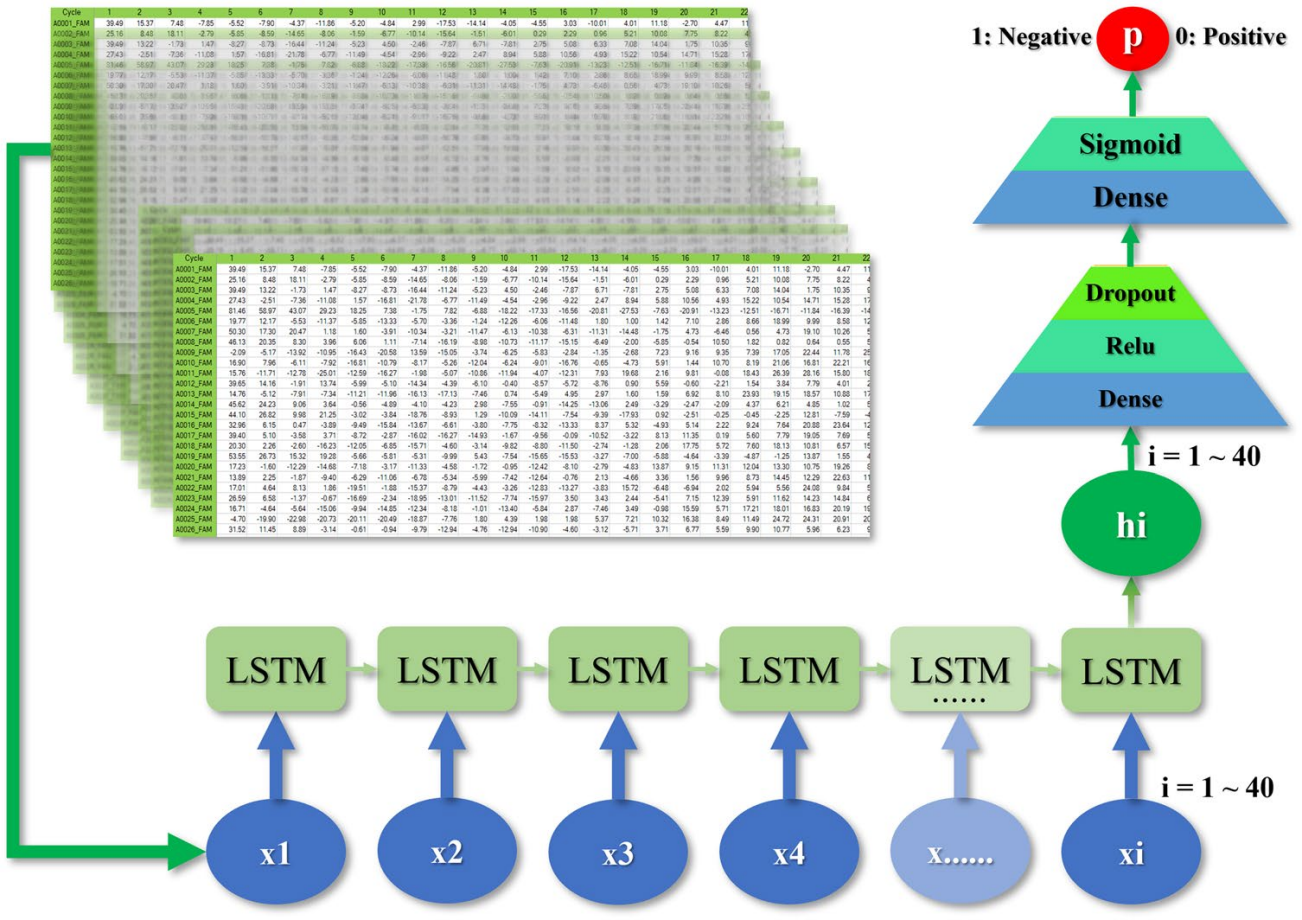
Development of the deep learning model. *LSTM* long short-term memory.

Since the fluorescence values derived in the RT-PCR process have the characteristics of time series data, we developed a total of 40 DL models using LSTM (Fig. 1). All deep learning analyses were performed using Python.

### Training and test datasets

**Figure 2:**
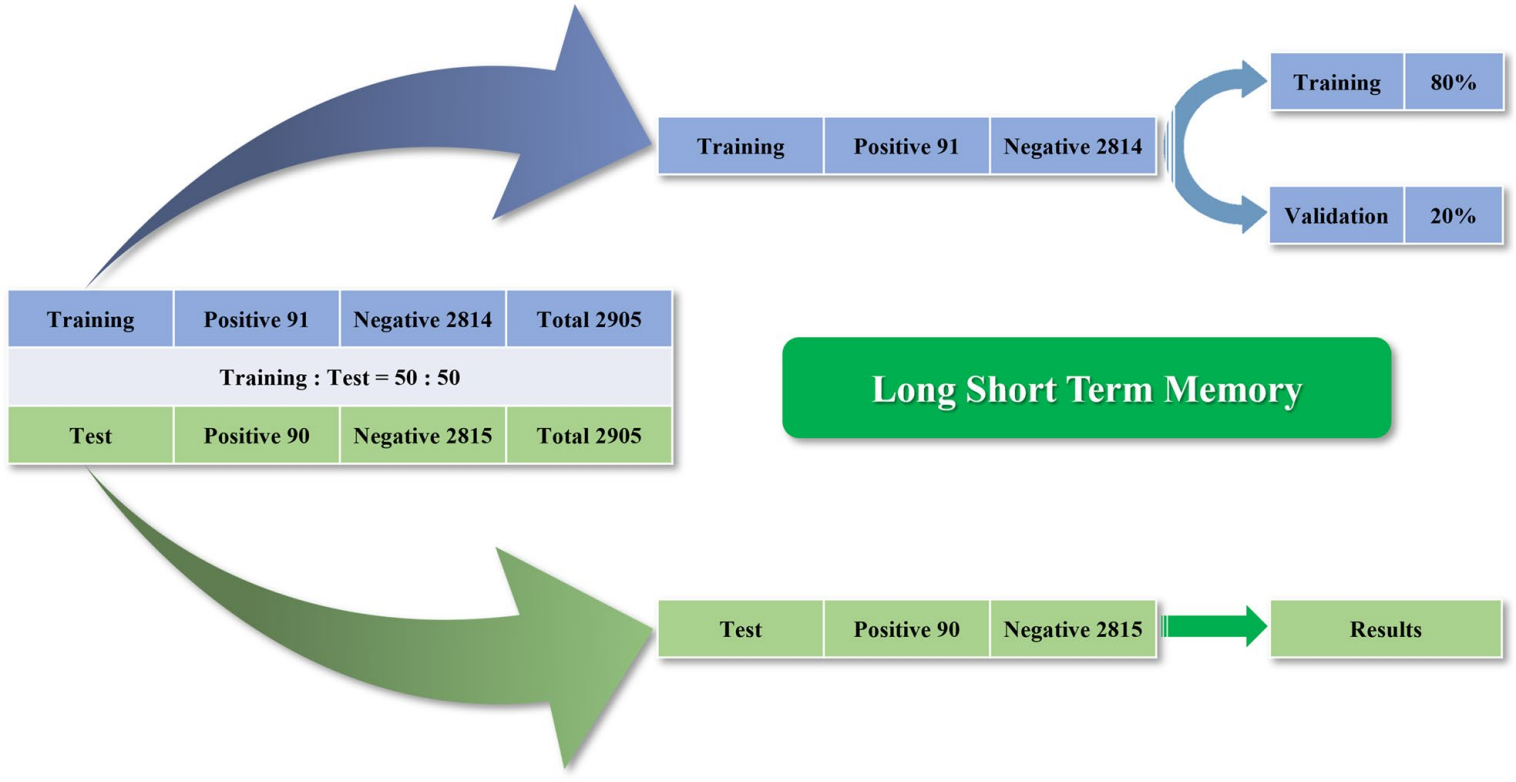
Composition of the training and test dataset.

The results of the RT-PCR virology test were used as the reference to train the models. Of the 5810 patients’ data included in the study, 181 had positive RT-PCR results, while 5629 had negative results. These data were divided into two datasets for training and testing. The data for training and validation were composed of curves of RT-PCR results of 91 positive cases and 2814 negative cases. The data of 90 positive and 2815 negative cases were used for testing (Fig. 2).

### Outcomes

Primary outcomes were the sensitivity, specificity and area under the receiver operating characteristic (AUROC) values of each model.

Secondary outcomes suggested an optimal model using positive predictive value (PPV), negative predictive value (NPV) and accuracy according to the prevalence of each model for several countries: the United States, Italy, and South Korea. The prevalence data for each country were referenced from the “COVID-19 Data Repository by the Center for Systems Science and Engineering (CSSE)” at Johns Hopkins University^11^. The prevalence was based on values measured for each country between June and July 2021. In a triangular-shaped radar chart using the PPV, NPV, and accuracy values affected by the prevalence to visualize diagnostic performance, each model was compared by calculating the ratio of the area of the triangle covered by each model to the total triangle area of the radar chart (Figs. 4, 5, 6).

### Statistical analysis

All statistical analyses were performed using SPSS software V.26.0 (IBM, SPSS, Inc., Chicago, IL, United States). Sensitivity (the proportion of true positives) and specificity (the proportion of true negatives) were calculated in comparison with the positivity or negativity of RT-PCR results. We calculated the false-positive and false-negative rates using the confusion matrix and calculated the PPVs and NPVs of each model using the COVID-19 prevalence for three countries: the United States, Italy and South Korea.

## Results

### Diagnostic performance of DL models

Table 1 shows the diagnostic performance of the data from the 20th model trained with raw data (1 to 20 cycles) to the 36th model trained with raw data (1 to 36 cycles). The sensitivity was the highest, at 100% (95% CI 95.98% to 100.0%), in the 33rd model and the 34th model, and the specificity was the highest, at 96.77% (95% CI 96.05% to 97.39%), in the 25th model. The AUROC value was the highest, at 97.00%, in the 33rd model, which had the highest sensitivity. The AUROC value of the 25th model, which had the highest specificity, was 88.38%. The model with the lowest false-positive rate (FPR) was the 25th model, at 3.23%, and the model with the lowest false-negative rate (FNR) was the 33rd model, at 0%.

**Table 1:** Diagnostic performance of deep learning models.

Model	Sensitivity, % (95% CI, %)	Specificity, %, (95% CI, %)	AUROC, %	False positive, %	False negative, %
20	76.53 (74.00 to 90.36)	68.56 (66.81 to 70.27)	75.95	31.44	16.67
21	93.33 (86.05 to 97.51)	75.72 (74.15 to 77.35)	84.55	24.23	6.67
22	66.67 (55.95 to 76.26)	89.38 (88.18 to 90.49)	78.02	10.62	33.33
23	83.33 (74.00 to 90.36)	92.40 (91.36 to 93.35)	87.87	7.60	16.67
24	90.00 (81.86 to 95.32)	92.54 (91.51 to 93.48)	91.27	7.46	10.00
25	80.00 (70.25 to 87.69)	96.77 (96.05 to 97.39)	88.38	3.23	20.00
26	92.22 (84.63 to 96.82)	91.69 (90.61 to 92.68)	91.95	8.31	7.78
27	93.33 (86.05 to 97.51)	92.58 (91.54 to 93.52)	92.95	7.42	6.67
28	96.67 (90.57 to 99.31)	93.25 (92.26 to 94.15)	94.96	6.75	3.33
29	94.44 (87.51 to 98.17)	85.04 (83.67 to 86.34)	89.74	14.96	5.56
30	96.67 (90.57 to 99.31)	91.08 (89.97 to 92.11)	93.88	8.92	3.33
31	97.78 (92.20 to 99.73)	90.55 (89.41 to 91.61)	94.16	9.45	2.22
32	96.67 (90.57 to 99.31)	92.68 (91.66 to 93.62)	94.67	7.32	3.33
33	100.00 (95.98 to 100.0)	94.00 (93.05 to 94.85)	97.00	6.00	0
34	100.00 (95.98 to 100.0)	93.32 (92.34 to 94.22)	96.66	6.68	0
35	97.78 (92.20 to 99.73)	95.52 (94.69 to 96.26)	96.65	4.48	2.22
36	98.89 (93.96 to 99.97)	93.07 (92.07 to 93.98)	95.98	6.93	1.11

*CI* confidence interval, *AUROC* area under the receiver operating characteristic curve.

### Comparison of the diagnostic performance of each model and RT-PCR

**Figure 3:**
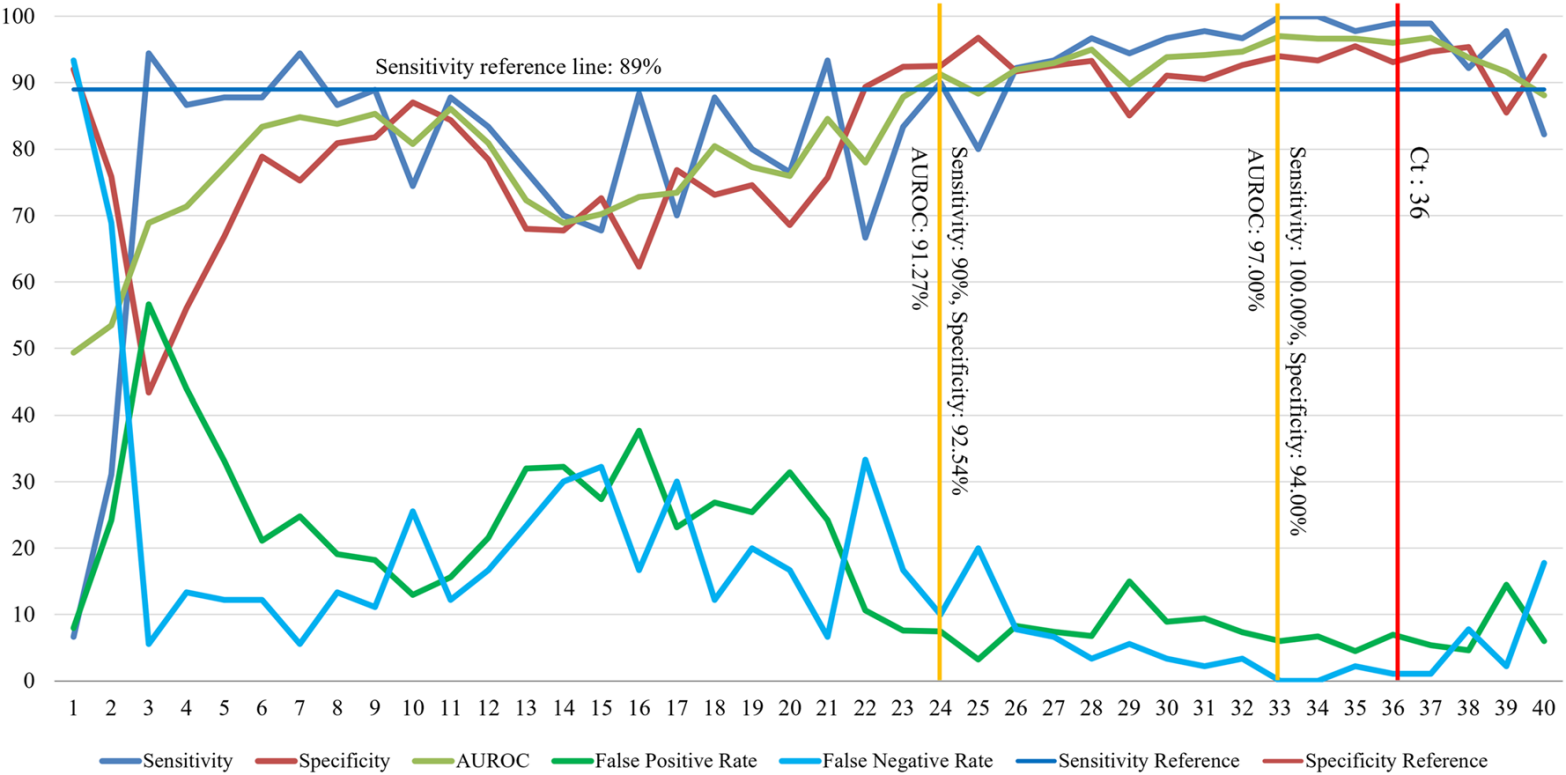
Comparison of the diagnostic performance of each model and RT-PCR. *AUROC* area under the receiver operating characteristic curve, *Ct* cycle threshold.

The test results of 40 DL models developed through LSTM are shown in Fig. 3. The sensitivity of each model was 93.33% (95% CI 86.05% to 97.51%) in the model constructed using raw data up to 21 cycles and considering the sensitivity reference value of 89% of RT-PCR. However, the 21st model had a low specificity of 75.72% (95% CI 74.15% to 77.35%). The 24th model showed sensitivity, specificity and AUROC values of 90% (95% CI 81.86% to 95.32%), 92.54% (95% CI 91.51% to 93.48%) and 91.27%, respectively (Fig. 3).

### Effects of prevalence on screening performance of each model: United States, Italy, South Korea

In the United States, showing a prevalence of 10.06%, the model with the highest positive predictive value (PPV) was the 25th model, at 73.33% (95% CI 68.67% to 77.53%), and the model with the highest negative predictive value (NPV) was the 33rd model, at 100% (95% CI N/A). Accuracy was the highest, at 95.75%, (95% CI 94.95 to 96.45), in the 35th model. The NPV of the 25th model with the highest PPV was 97.76% (95% CI 96.64% to 98.50%), and the accuracy was 95.09% (95% CI 94.24% to 95.85%). The PPV of the 33rd model with the highest NPV was 64.92% (95% CI 61.52% to 68.17%), and the accuracy was 94.60% (95% CI 93.71% to 95.39%) (Table 2).

**Table 2:** Effects of prevalence on screening performance of each model: United States (prevalence, 10.06%).

Model	PPV, % (95% CI, %)	NPV, % (95% CI, %)	Accuracy, % (95% CI, %)
20	21.29 (20.92 to 24.69)	96.34 (95.89 to 98.33)	69.36 (68.34 to 71.70)
21	29.93 (28.21 to 31.80)	99.03 (97.93 to 99.55)	77.48 (75.97 to 79.04)
22	41.09 (36.78 to 45.53)	96.02 (94.74 to 97.00)	87.11 (85.83 to 88.31)
23	54.91 (50.97 to 58.80)	98.04 (96.92 to 98.75)	91.49 (90.42 to 92.48)
24	57.27 (53.64 to 60.83)	98.81 (97.82 to 99.36)	92.29 (91.26 to 93.23)
25	73.33 (68.67 to 77.53)	97.76 (96.64 to 98.50)	95.09 (94.24 to 95.85)
26	55.21 (51.81 to 58.56)	99.07 (98.12 to 99.54)	91.74 (90.68 to 92.72)
27	58.28 (54.80 to 61.68)	99.21 (98.30 to 99.63)	92.65 (91.64 to 93.57)
28	61.41 (57.98 to 64.73)	99.60 (98.81 to 99.87)	93.59 (92.64 to 94.46)
29	41.23 (38.80 to 43.71)	99.28 (98.33 to 99.69)	85.98 (84.67 to 87.23)
30	54.64 (51.55 to 57.70)	99.60 (98.78 to 99.87)	91.64 (90.58 to 92.62)
31	53.48 (50.53 to 56.42)	99.73 (98.94 to 99.93)	91.27 (90.19 to 92.27)
32	59.48 (56.14 to 62.73)	99.60 (98.80 to 99.87)	93.08 (92.10 to 93.98)
33	64.92 (61.52 to 68.17)	100.00 (N/A**)**	94.60 (93.71 to 95.39)
34	62.46 (59.17 to 65.64)	100.00 (N/A)	93.99 (93.06 to 94.83)
35	70.82 (67.11 to 74.27)	99.74 (98.99 to 99.93)	95.75 (94.95 to 96.45)
36	61.33 (58.03 to 64.53)	99.87 (99.08 to 99.98)	93.65 (92.71 to 94.51)

*CI* confidence interval, *PPV* positive predictive value, *NPV* negative predictive value, *N/A* not applicable.

In Italy, which showed a prevalence of 6.98%, the model with the highest PPV was the 25th model, at 65.02% (98% CI 59.68% to 69.97%), and the model with the highest NPV was the 33rd model, at 100% (95% CI N/A). The accuracy was the highest, at 95.68% (95% CI 94.88% to 96.39%), in the 35th model. The NPV of the 25th model with the highest PPV was 98.47% (95% CI 97.71% to 98.98%), and the accuracy was 95.60% (95% CI 94.79% to 96.31%). The PPV of the 33rd model with the highest NPV was 55.57% (95% CI 51.92% to 59.13%), and the accuracy was 94.42% (95% CI 93.52% to 95.22%) (Table 3).

**Table 3:** Effects of prevalence on screening performance of each model: Italy (prevalence, 6.98%).

Model	PPV, % (95% CI, %)	NPV, % (95% CI, %)	Accuracy, % (95% CI, %)
20	16.60 (15.16 to 18.13)	98.21 (97.18 to 98.86)	69.59 (67.88 to 71.26)
21	22.44 (20.97 to 23.95)	99.34 (98.59 to 99.70)	77.00 (75.42 to 78.52)
22	32.03 (28.21 to 36.08)	97.28 (96.39 to 97.95)	87.79 (86.55 to 88.96)
23	45.15 (41.24 to 49.08)	98.66 (97.90 to 99.15)	91.76 (90.71 to 92.74)
24	47.53 (43.86 to 51.19)	99.20 (98.52 to 99.57)	92.36 (91.34 to 93.30)
25	65.02 (59.68 to 69.97)	98.47 (97.71 to 98.98)	95.60 (94.79 to 96.31)
26	45.45 (42.07 to 48.83)	99.37 (98.72 to 99.69)	91.72 (90.66 to 92.70)
27	48.56 (45.02 to 52.08)	99.46 (98.84 to 99.75)	92.63 (91.62 to 93.55)
28	51.82 (48.24 to 55.34)	99.73 (99.19 to 99.91)	93.49 (92.53 to 94.36)
29	32.17 (29.98 to 34.40)	99.51 (98.86 to 99.79)	85.70 (84.37 to 86.95)
30	44.88 (41.81 to 47.94)	99.73 (99.17 to 99.91)	91.74 (90.40 to 92.46)
31	43.73 (40.82 to 46.64)	99.82 (99.28 to 99.95)	91.06 (89.96 to 92.07)
32	49.80 (46.36 to 53.20)	99.73 (99.19 to 99.91)	92.96 (91.97 to 93.86)
33	55.57 (51.92 to 59.13)	100.00 (N/A)	94.42 (93.52 to 95.22)
34	52.93 (49.46 to 56.33)	100.00 (N/A)	93.79 (92.85 to 94.64)
35	61.13 (57.95 to 66.10)	99.83 (99.32 to 99.96)	95.68 (94.88 to 96.39)
36	51.72 (48.29 to 55.13)	99.91 (99.37 to 99.99)	93.48 (92.52 to 94.35)

*CI* confidence interval, *PPV* positive predictive value, *NPV* negative predictive value, *N/A* not applicable.

In South Korea, which showed a prevalence of 0.27%, the model with the highest PPV was the 25th model, at 6.43% (95% CI 5.07% to 7.76%), and the model with the highest NPV was the 33rd model, at 100% (95% CI N/A). Accuracy was the highest, at 96.72% (95% CI 96.01% to 97.34%), in the 25th model. The NPV of the 25th model with the highest PPV was 99.94% (95% CI 99.92% to 99.96%). The PPV of the 33rd model with the highest NPV was 4.42% (95% CI 3.75% to 4.96%), and the accuracy was 94.01% (95% CI 93.09% to 94.85%) (Table 4).

**Table 4:** Effects of prevalence on screening performance of each model: South Korea (prevalence, 0.27%).

Model	PPV, % (95% CI, %)	NPV, % (95% CI, %)	Accuracy, % (95% CI, %)
20	0.67 (0.64 to 0.79)	99.91 (99.90 to 99.96)	68.58 (66.88 to 70.29)
21	1.06 (0.95 to 1.12)	99.98 (99.95 to 99.99)	75.77 (74.22 to 77.37)
22	1.71 (1.40 to 2.00)	99.90 (99.86 to 99.92)	89.32 (88.14 to 90.42)
23	2.95 (2.47 to 3.36)	99.95 (99.92 to 99.97)	92.37 (91.35 to 93.31)
24	3.24 (2.74 to 3.65)	99.97 (99.95 to 99.98)	92.53 (91.52 to 93.46)
25	6.43 (5.07 to 7.76)	99.94 (99.92 to 99.96)	96.72 (96.01 to 97.34)
26	2.99 (2.55 to 3.33)	99.98 (99.95 to 99.99)	91.69 (90.63 to 92.67)
27	3.37 (2.87 to 3.77)	99.98 (99.96 to 99.99)	92.58 (91.56 to 93.50)
28	3.83 (3.25 to 4.28)	99.99 (99.97 to 100.00)	93.26 (92.29 to 94.14)
29	1.72 (1.52 to 1.86)	99.98 (99.96 to 99.99)	85.07 (83.72 to 86.35)
30	2.92 (2.53 to 3.22)	99.99 (99.97 to 100.00)	91.10 (90.00 to 92.11)
31	2.79 (2.43 to 3.06)	99.99 (99.97 to 100.00)	90.57 (89.45 to 91.61)
32	3.54 (3.02 to 3.94)	99.99 (99.97 to 100.00)	92.69 (91.69 to 93.61)
33	4.42 (3.75 to 4.96)	100.00 (N/A)	94.01 (93.09 to 94.85)
34	3.99 (3.41 to 4.45)	100.00 (N/A)	93.34 (92.37 to 94.22)
35	5.72 (4.74 to 6.57)	99.99 (99.98 to 100.00)	95.53 (94.71 to 96.25)
36	3.72 (3.26 to 4.24)	100 (99.98 to 100.00)	93.09 (92.10 to 93.98)

*CI* confidence interval, *PPV* positive predictive value, *NPV* negative predictive value, *N/A* not applicable.

### Comparison of each model considering the effects of prevalence on screening performance: United States, Italy, South Korea

**Figure 4:**
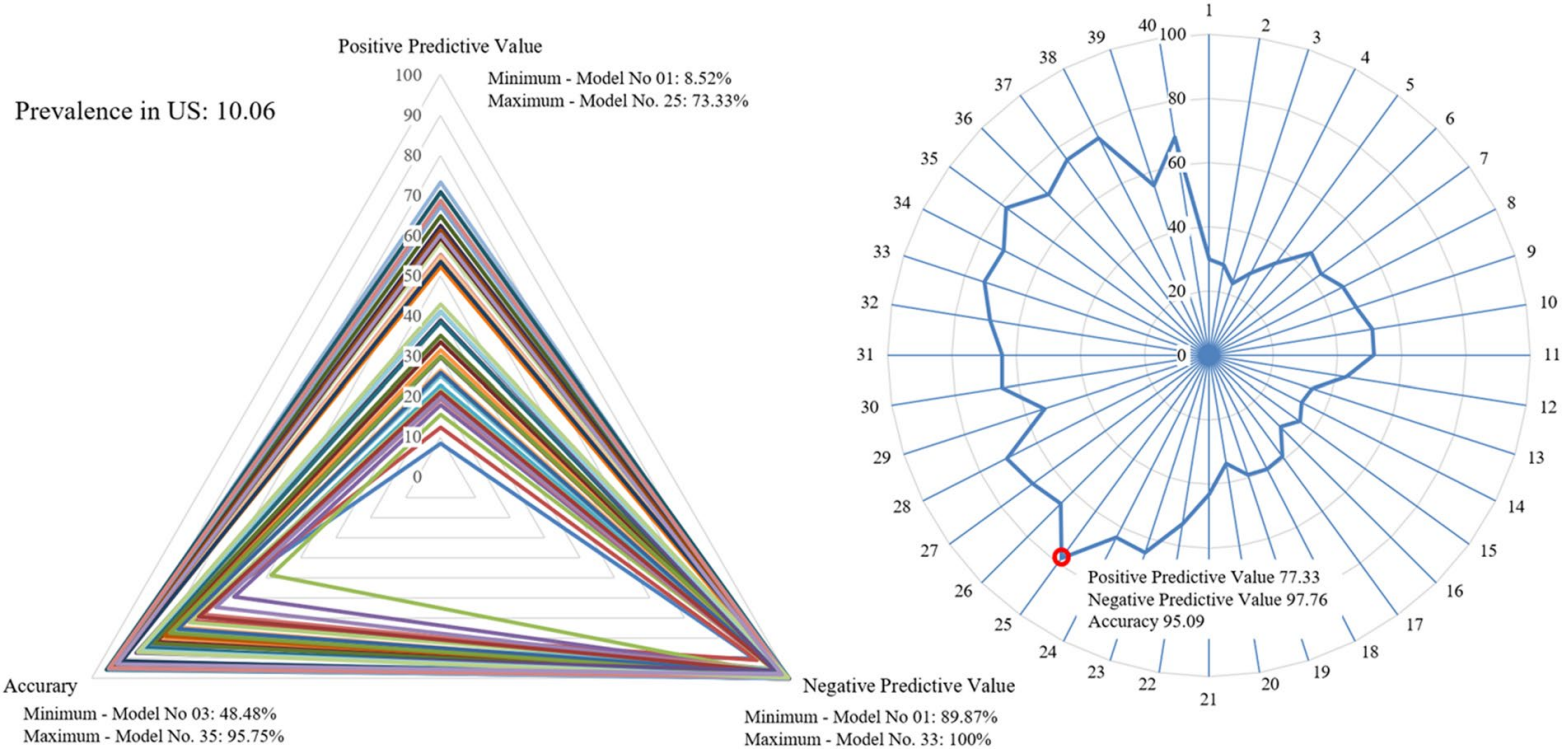
Comparison of each model considering screening performance affected by prevalence: United States. Triangle radar chart: positive predictive values, negative predictive values and accuracy of each model; polygonal radar chart: triangle radar chart area ratio in each model.

In the United States, which showed a prevalence of 10.06%, the model with the lowest PPV was the 1st model, at 8.52% (95% CI 4.08% to 16.92%), and the highest PPV was that of the 25th model, at 73.33% (95% CI 68.67% to 77.53%). The model with the lowest NPV was the 1st model, at 89.87% (95% CI 89.35% to 90.37%), and the highest PPV was that of the 33rd model, at 100% (95% CI N/A). Accuracy was the lowest at 48.48% (95% CI 46.65% to 50.32%) in the 3rd model and the highest at 95.75% (95% CI 94.95% to 96.45%) in the 35th model. The 25th model was the model with the largest proportion of area occupied by the radar chart, at 78.13% (95% CI 74.05% to 79.96%) (Fig. 4).

**Figure 5:**
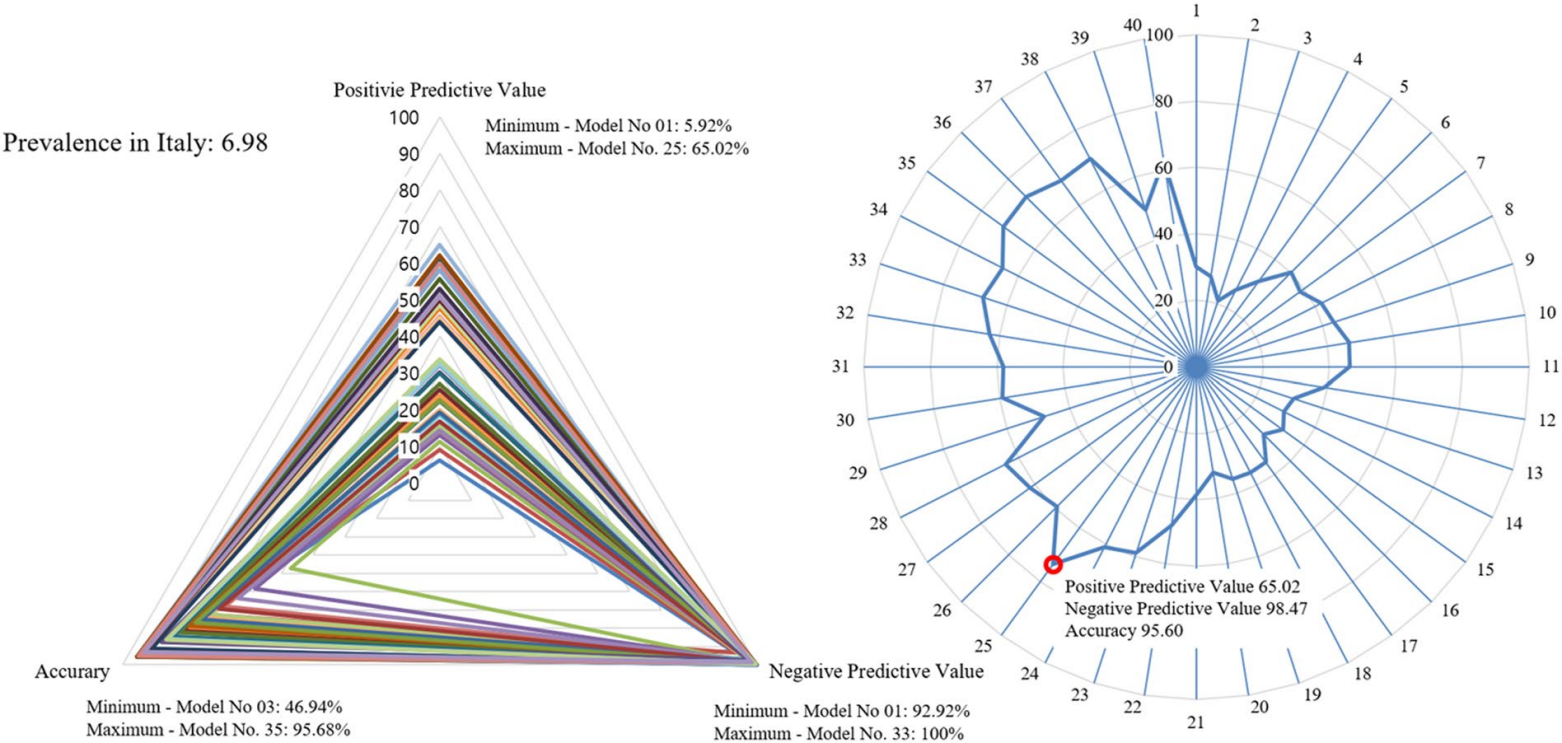
Comparison of each model considering screening performance affected by prevalence: Italy. Triangle radar chart: positive predictive values, negative predictive values and accuracy of each model; polygonal radar chart: triangle radar chart area ratio in each model.

In Italy, which showed a prevalence of 6.98%, the model with the lowest PPV was the 1st model, at 5.92% (95% CI 2.79% to 12.09%), and the highest PPV was that of the 25th model, at 65.02% (95% CI 59.68% to 69.97%). The model with the lowest NPV was the 1st model, at 92.92% (95% CI 92.55% to 93.29%), and the highest NPV was that of the 33rd model, at 100% (95% CI N/A). Accuracy was the lowest, at 46.94% (95% CI 45.11% to 48.77%), in the 3rd model and the highest, at 95.68% (95% CI 94.88% to 96.39%), in the 35th model. The 25th model was the model with the largest proportion of area occupied by the radar chart, at 73.44% (95% CI 69.17% to 77.32%) (Fig. 5).

**Figure 6:**
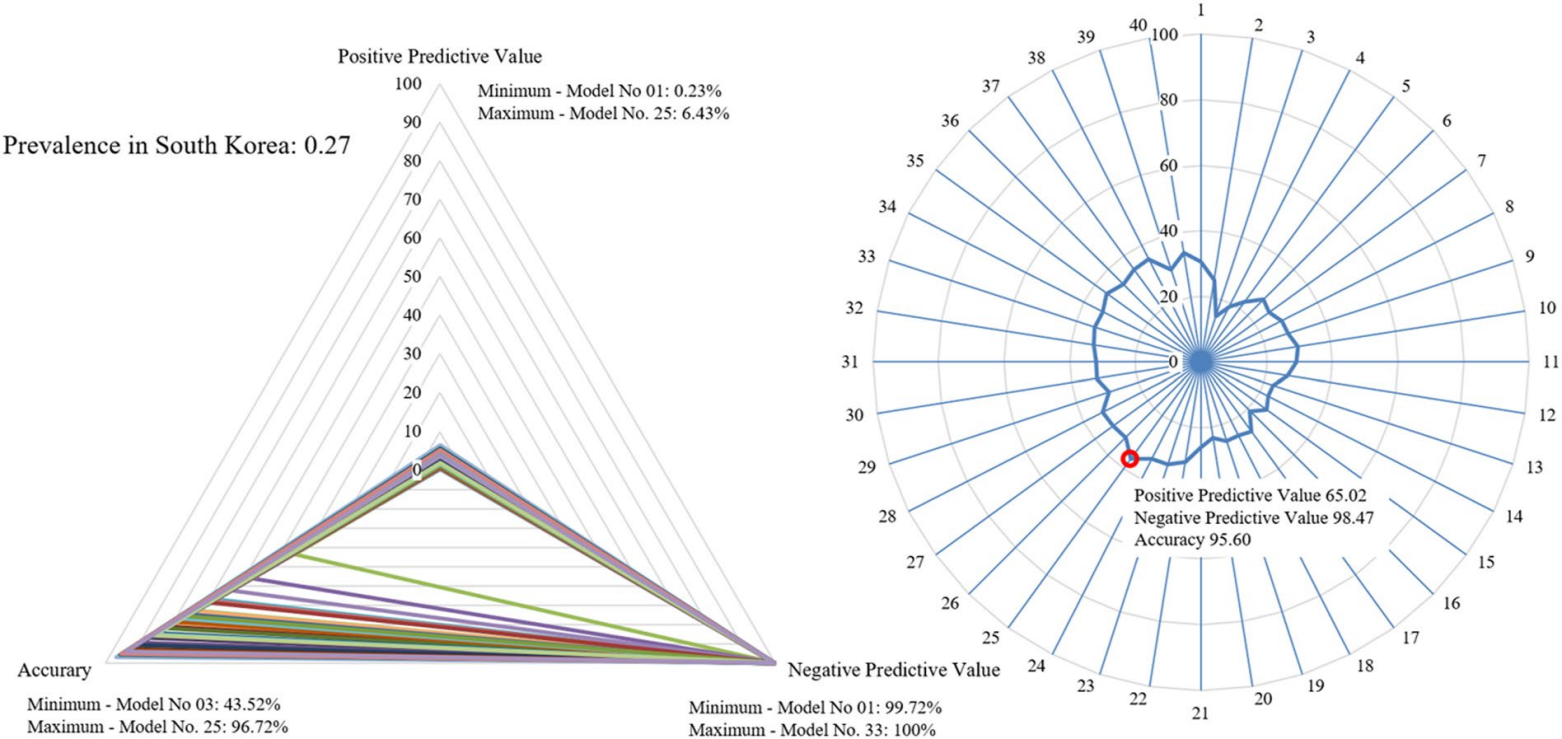
Comparison of each model considering screening performance affected by prevalence: South Korea. Triangle radar chart: positive predictive values, negative predictive values and accuracy of each model; polygonal radar chart: triangle radar chart area ratio in each model.

In South Korea, which showed a prevalence of 0.27%, the model with the lowest PPV was the 1st model, at 0.23% (95% CI 0.10% to 0.49%), and the highest PPV was that of the 25th model, at 6.43% (95% CI 5.07% to 7.76%). The model with the lowest NPV was the 1st model, at 99.72% (95% CI 99.71% to 99.74%), and the highest NPV was that of the 33rd model, at 100% (95% CI N/A). Accuracy was the lowest, at 43.52% (95% CI 41.70% to 45.34%), in the 3rd model and the highest, at 96.72% (95% CI 96.01% to 97.34%), in the 25th model. The 25th model was the model with the largest proportion of area occupied by the radar chart, at 36.44% (95% CI 35.29% to 37.54%) (Fig. 6).

## Discussion

In this study, we developed a total of 40 DL models to reduce the time required for the diagnosis of COVID-19 using RT-PCR as much as possible and compared the diagnostic and screening performance of each model.

In a previous meta-analysis, Kim et al.^12^ determined that the pooled sensitivity of RT-PCR was 89%, and the PPVs and NPVs, affected by the prevalence, were 47.3% to 98.3% and 93.4% to 99.9%, respectively. We used the pooled sensitivity of the RT-PCR test investigated by Kim et al. to compare the performance of each model obtained in this study as a reference value.

Considering a pooled RT-PCR sensitivity of 89% as a sensitivity reference value^12^, the sensitivity of the 21st model exceeded this standard, at 93.33% (95% CI 86.05% to 97.51%). In addition, considering the approximate trend of diagnostic performance of all models, the 24th model, with a sensitivity of 90% (95% CI 81.86% to 95.32%), showed a tendency to exceed the sensitivity reference value (Fig. 3). In view of these results, using a Ct value of 36 rather than the time taken by 40 cycles for RT-PCR diagnosis, it can be inferred that a meaningful time reduction may be possible through the development of this DL model.

Furthermore, the sensitivity reference value was exceeded or showed a similar level from the 3rd model to the 9th model and in the 11th, 16th, and 18th models (Supplementary Table 1). However, the specificities of these models were generally lower than 80%, so it was difficult to judge whether the model was appropriate based on the diagnostic performance.

In the case of the PPV in this study, in the United States, where the prevalence was 10.06%, the 25th model showed the highest PPV at 73.33%. Similarly, in Italy, with a prevalence of 6.98%, and South Korea, with a prevalence of 0.27%, the PPV was highest in the same model as that in the United States, at 65.02% and 6.43%, respectively (Tables 2, 3, 4). However, according to the study results of Kim et al.^12^, in the United States, with a prevalence of 17.7% in March–April 2020; Germany, with a prevalence of 5.7%; and Taiwan, with a prevalence of 1%; the PPVs of RT-PCR itself were 95%, 84.3% and 47.3%, respectively. Although the prevalence did not match between the two studies and the timing at which the prevalence was measured was different, considering the range of prevalence levels, it can be inferred that the positive screening performance of the model developed in this study is somewhat inferior to that of RT-PCR.

On the other hand, in the case of negative screening performance, which is affected by the prevalence, in the United States, where the prevalence is 10.06%, the 20th model showed an NPV of 96.34% (95% CI 95.89% to 98.33%), and in Italy (prevalence 6.98%) and South Korea (prevalence 0.27%), the NPVs were 98.21% (95% CI 97.18% to 98.86%) and 99.21% (95% CI 99.90% to 99.96%) in the same model, respectively. These findings show that the negative screening performance of the model developed using fluorescence values up to 20 cycles, which is half of the 40 cycles, is very good (Tables 2, 3, 4).

Furthermore, in research reported by Kim et al.^12^, the PPV and NPV of RT-PCR showed a distribution of 47.3% to 98.3% and 93.4% to 99.9%, respectively, according to the national prevalence (prevalence range of 1% to 39% from March to April 2020). The negative screening performance of the models developed in this study can be considered at a similar level to that of RT-PCR. Although the statistical significance cannot be compared, this result shows that the model trained only with raw data up to 20 cycles differs little from the negative screening performance of RT-PCR itself, for which all 40 cycles were evaluated.

In this study, we created a radar chart for each model using PPV, NPV and accuracy, which were affected by prevalence, representing screening performance (Figs. 4, 5, 6). Then, the screening performance of each model was expressed as the ratio of the area covered by each model to the total area of the radar chart as a percentage, and the area ratio of each model was entered into a radar chart. This chart confirmed that the model with the largest area ratio was the 25th model when considering the PPV, NPV and accuracy. We propose that it would be reasonable to present the 25th model as a model with minimal bias in negative screening performance, positive screening performance and accuracy based on these results.

To the best of our knowledge, no study has reduced the time required to diagnose based on RT-PCR by developing a model trained with raw RT-PCR data and confirming its diagnostic performance. In addition, since the start of the COVID-19 pandemic, no similar research design has been reported in papers that reviewed the performance of various artificial intelligence or deep learning models for diagnosing COVID-19 until recently^13^. Although there was a single study that used RT-PCR curves to build an AI model such as a convolutional neural network (CNN) to reduce false-positive diagnoses, the study was not related to shortening the time for diagnosis and used graph images, differentiating it from our study^14^.

In addition, a recently published AI- and DL-related COVID-19 diagnostic study presented a model trained on CT images or CXR images using various CNN methods. Other studies on the diagnosis of COVID-19 have reported on models trained with blood test results or clinical information. First, in the studies that reported the performance of models trained based on CNNs using chest CT images, the sensitivity ranged from 77 to 90%, the specificity ranged from 68 to 96.6%, and the AUROC ranged from 0.85 to 0.97^1–3,15–20^. Second, in studies that reported the performance of models trained on CNNs using chest CXR images, the sensitivity ranged from 78 to 97%, the specificity ranged from 72.6 to 99.17%, and the AUROC ranged from 0.77 to 0.92^4–7,21–23^. Third, there have been studies evaluating the diagnostic performance of COVID-19 using models trained with blood tests or clinical information. In these studies, the sensitivity ranged from 66 to 93%, the specificity ranged from 64 to 97.9%, and the AUROC ranged from 0.86 to 0.979^24–26^. Considering the diagnostic performance of the various models presented in these references, the diagnostic performance of the model developed in this study appears to be sufficiently high.

What is needed in the clinical field is to increase the efficiency of hospital bed resource management through rapid isolation, rapid diagnosis, and rapid and safe release from isolation. From that perspective, the above studies suggest that COVID-19 diagnosis may be possible through the application of AI. Nevertheless, the models presented in the existing references have lower clinical relevance when considering the realistic clinical conditions due to the following problems.

Due to the imbalance and bias of the data selected for use in training, we question whether this approach can be safely used in clinical settings for the diagnosis of COVID-19. On these issues, Laghi A agrees that efforts to diagnose COVID-19 through AI models are necessary. However, he noted that it seems very risky to trust the diagnostic performance of the AI models presented in these studies and use it in clinical settings because imaging tests such as CXR or chest CT at the early stage of COVID-19 infection can show normal findings^27^.

The model developed in the present study is not trained from imaging tests such as CXR or chest CT, blood test results, or clinical information, as in previous studies. In this study, a model trained with LSTM was developed as a DL method applied to time series data training using raw data from 1 to 40 cycles of RT-PCR. Thus, there is potential for early diagnosis via RT-PCR using the DL model developed in this study.

In this study, the sensitivity of the 21st model started to exceed the sensitivity reference value, and the sensitivity and specificity of the 24th model exceeded a sensitivity of 90% (Table 1, Fig. 3). Considering the time it takes to diagnose RT-PCR, the diagnostic performance of the model developed in this study shows the possibility of reducing the time required for RT-PCR diagnosis by almost half.

In addition, the model developed in this study showed that the PPV had somewhat lower positive screening performance than RT-PCR; however, the NPV showed negative screening performance similar to that of RT-PCR (Tables 2, 3, 4). Considering this excellent negative screening performance, if various information, such as the patient's clinical characteristics, blood test results, and imaging information, such as CXR or chest CT results, are combined with this DL model, it can be assumed that the diagnostic performance for early diagnosis will be improved. We can infer that employing this model has the potential to contribute to improving the efficiency of in-hospital bed resource management for patients with fever or screening symptoms.

This study has several limitations:

First, 181 positive cases and 5629 negative cases used for training constituted too few positive cases compared to negative cases. This data bias can affect the diagnostic performance of the developed DL models, and in the end, it is difficult to apply the DL model universally. However, through this study, we were able to confirm that the diagnostic performance was not significantly impaired by not performing all 40 cycles of PCR.

Second, other than LSTM, other DL methods that can be trained using time series data were not applied. As a result, it is not known whether LSTM is the best method because comparative analysis with models that can be developed through other DL methods has not been performed. Nevertheless, LSTM is an RNN-based method that was first selected and used in this study because this method was developed to solve the vanishing gradient problem of existing RNNs^28^. Of course, it is necessary to collect additional data in a follow-up study and perform comparative analysis with DL methods applied to time series data.

Third, a range of evaluation metrics were not used in this study. As described in the second limitation, the proposed model could not be compared with models developed through other methods. We acknowledge that it is difficult to apply evaluation methods other than showing the level of diagnostic performance of the model with this study design. Understanding this limitation, we paid attention to the difference in prevalence by country, investigated the screening performance of the model for each representative country, and presented the results.

Fourth, the method of presenting the screening performance of the model as the ratio of the area of the radar chart is not generally employed. The area of the triangle is calculated assuming the PPV, NPV, and accuracy to have a 1:1:1 weight ratio. Therefore, if this weight ratio is set differently, that is, if the three weights are set differently according to need (such as accuracy being more important, etc.), the calculated area and the ratio may be different. Nevertheless, as the PPV, NPV and accuracy all have high values, it is natural that the screening power is high. We believe that the ratio of the area of the radar chart does not perfectly reflect the screening power of the DL model; however, it does help to explain the approximate trend.

## Conclusion

Through the test results of the DL models developed in this study, we confirmed the possibility of shortening the diagnosis time of RT-PCR without impairing its diagnostic performance. This reduction in time to diagnosis is expected to be of great help in managing insufficient bed resources in the clinical field.

## Supplementary Information

Supplementary Tables 1.

Supplementary Information 2.

Supplementary Information 3.

## Data Availability

All data generated or analysed during this study are included in this published article (and its supplementary information files).
