# Supplementary material for: The application of a deep learning system developed to reduce the time for RT-PCR in COVID-19 detection
Source: Sci Rep. 2022 Jan 24;12:1234. doi: 10.1038/s41598-022-05069-2 (PMC8786863; doi:10.1038/s41598-022-05069-2)
Supplement: Supplementary file 1 — Supplementary Tables 1. [file 41598_2022_5069_MOESM1_ESM.docx]

Supplementary Table 1. Diagnostic performance in each model

| Model No. | Sensitivity, % (95% CI; %) | Specificity, %, (95% CI; %) | AUROC, % | False Positive, % | False Negative, % |
| --- | --- | --- | --- | --- | --- |
| 1 | 6.67(2.49 to 13.95) | 92.04(90.98 to 93.02) | 49.35 | 7.96 | 93.33 |
| 2 | 31.11(21.77 to 41.74) | 75.81(74.18 to 77.3) | 53.46 | 24.19 | 68.89 |
| 3 | 94.44(87.51 to 98.17) | 43.37(41.53 to 45.23) | 68.91 | 56.63 | 5.56 |
| 4 | 86.67(77.87 to 92.92) | 56.09(54.24 to 57.94) | 71.38 | 43.91 | 13.33 |
| 5 | 87.78(79.18 to 93.74) | 66.86(65.08 to 68.59) | 77.32 | 33.14 | 12.22 |
| 6 | 87.78(79.18 to 93.74) | 78.90(77.34 to 80.39) | 83.34 | 21.10 | 12.22 |
| 7 | 94.44(87.51 to 98.17) | 75.24(73.60 to 76.83) | 84.84 | 24.76 | 5.56 |
| 8 | 86.67(77.87 to 92.92) | 80.92(79.42 to 82.36) | 83.80 | 19.08 | 13.33 |
| 9 | 88.89(80.51 to 94.54) | 81.78(80.30 to 83.19) | 85.33 | 18.22 | 11.11 |
| 10 | 74.44(64.16 to 83.06) | 87.03(85.74 to 88.25) | 80.74 | 12.97 | 25.25 |
| 11 | 87.78(79.18 to 93.74) | 84.40(83.01 to85.73) | 86.09 | 15.6 | 12.22 |
| 12 | 83.33(74.00 to 90.36) | 78.44(76.87 to 79.94) | 80.89 | 21.56 | 16.67 |
| 13 | 76.67(66.57 to 84.94) | 67.99(66.23 to 69.71) | 72.33 | 32.01 | 23.33 |
| 14 | 70.00(59.43 to 79.21) | 67.78(66.02 to 69.50) | 68.89 | 32.22 | 30 |
| 15 | 67.78(57.10 to 77.25) | 72.61(70.92 to 74.25) | 70.19 | 27.39 | 32.22 |
| 16 | 88.33(74.00 to 90.36) | 62.31(60.49 to 64.10) | 72.82 | 37.69 | 16.67 |
| 17 | 70.00(59.43 to 79.21) | 76.87(75.27 to 78.42) | 73.44 | 23.13 | 30 |
| 18 | 87.78(79.18 to 93.74) | 73.11(71.43 to 74.74) | 80.44 | 26.89 | 12.22 |
| 19 | 80.00(70.25 to 87.69) | 74.60(72.95 to 76.20) | 77.30 | 25.40 | 20 |
| 20 | 76.53 (74.00 to 90.36) | 68.56 (66.81 to 70.27) | 75.95 | 31.44 | 16.67 |
| 21 | 93.33 (86.05 to 97.51) | 75.72 (74.15 to 77.35) | 84.55 | 24.23 | 6.67 |
| 22 | 66.67 (55.95 to 76.26) | 89.38 (88.18 to 90.49) | 78.02 | 10.62 | 33.33 |
| 23 | 83.33 (74.00 to 90.36) | 92.40 (91.36 to 93.35) | 87.87 | 7.60 | 16.67 |
| 24 | 90.00 (81.86 to 95.32) | 92.54 (91.51 to 93.48) | 91.27 | 7.46 | 10.00 |
| 25 | 80.00 (70.25 to 87.69) | 96.77 (96.05 to 97.39) | 88.38 | 3.23 | 20.00 |
| 26 | 92.22 (84.63 to 96.82) | 91.69 (90.61 to 92.68) | 91.95 | 8.31 | 7.78 |
| 27 | 93.33 (86.05 to 97.51) | 92.58 (91.54 to 93.52) | 92.95 | 7.42 | 6.67 |
| 28 | 96.67 (90.57 to 99.31) | 93.25 (92.26 to 94.15) | 94.96 | 6.75 | 3.33 |
| 29 | 94.44 (87.51 to 98.17) | 85.04 (83.67 to 86.34) | 89.74 | 14.96 | 5.56 |
| 30 | 96.67 (90.57 to 99.31) | 91.08 (89.97 to 92.11) | 93.88 | 8.92 | 3.33 |
| 31 | 97.78 (92.20 to 99.73) | 90.55 (89.41 to 91.61) | 94.16 | 9.45 | 2.22 |
| 32 | 96.67 (90.57 to 99.31) | 92.68 (91.66 to 93.62) | 94.67 | 7.32 | 3.33 |
| 33 | 100.00 (95.98 to 100.0) | 94.00 (93.05 to 94.85) | 97.00 | 6.00 | 0 |
| 34 | 100.00 (95.98 to 100.0) | 93.32 (92.34 to 94.22) | 96.66 | 6.68 | 0 |
| 35 | 97.78 (92.20 to 99.73) | 95.52 (94.69 to 96.26) | 96.65 | 4.48 | 2.22 |
| 36 | 98.89 (93.96 to 99.97) | 93.07 (92.07 to 93.98) | 95.98 | 6.93 | 1.11 |
| 37 | 98.89(93.96 to 99.97) | 94.64(93.74 to 95.44) | 96.76 | 5.36 | 1.11 |
| 38 | 92.22(84.63 to 96.82) | 95.35(94.50 to 96.09) | 93.78 | 4.65 | 7.78 |
| 39 | 97.78(92.20 to 99.73) | 85.54(84.19 to 86.82) | 91.66 | 14.46 | 2.22 |
| 40 | 82.22(72.74 to 89.48) | 94.00(93.05 to 94.85) | 88.11 | 6.00 | 17.80 |

Supplementary Table 2. Screening performance of each deep learning model according to prevalence of US (10.06%)

| Model No. | PPV, % (95% CI; %) | NPV, % (95% CI; %) | Accuracy, % (95% CI, %) |
| --- | --- | --- | --- |
| 1 | 8.52(4.08 to 16.92) | 89.87(89.35 to 90.37) | 83.51(82.10 to 84.84) |
| 2 | 12.5(9.45 to 16.36) | 90.83(89.59 to 91.93) | 71.34(69.66 to 72.98) |
| 3 | 15.63(14.86 to 16.44) | 98.60(96.77 to 99.40) | 48.48(46.65 to 50.32) |
| 4 | 17.99(16.68 to 19.37) | 97.43(95.71 to 98.47) | 59.15(57.34 to 60.94) |
| 5 | 22.74(21.14 to 24.42) | 98.01(96.58 to 98.85) | 68.95(67.23 to 70.63) |
| 6 | 31.61(29.38 to 33.92) | 98.31(97.09 to 99.02) | 79.79(78.28 to 81.23) |
| 7 | 29.77(28.09 to 31.50) | 99.19(98.11 to 99.65) | 77.16(75.59 to 78.68) |
| 8 | 33.55(31.11 to 36.07) | 98.20(96.99 to 98.93) | 81.50(80.04 to 82.89) |
| 9 | 35.15(32.75 to 37.62) | 98.51(97.36 to 99.17) | 82.49(81.06 to 83.85) |
| 10 | 38.95(35.35 to 42.67) | 96.84(95.56 to 97.76) | 85.77(84.45 to 87.03) |
| 11 | 38.48(35.78 to 41.24) | 98.42(97.28 to 99.08) | 84.74(83.38 to 86.03) |
| 12 | 30.04(27.66 to 32.54) | 97.69(96.39 to 98.53) | 78.93(77.40 to 80.40) |
| 13 | 21.02(19.00 to 23.19) | 96.33(94.74 to 97.45) | 68.86(67.14 to 70.54) |
| 14 | 19.45(17.27 to 21.83) | 95.31(93.68 to 96.54) | 68.00(66.27 to 69.70) |
| 15 | 21.57(19.07 to 24.30) | 95.30(93.76 to 96.48) | 72.13(70.46 to 73.75) |
| 16 | 19.72(18.13 to 21.42) | 97.11(95.49 to 98.16) | 64.41(62.64 to 66.15) |
| 17 | 25.17(22.43 to 28.12) | 95.84(94.39 to 96.94) | 76.19(74.59 to 77.73) |
| 18 | 26.62(24.74 to 28.58) | 98.18(96.87 to 98.94) | 74.58(72.95 to 76.15) |
| 19 | 25.92(23.67 to 28.32) | 97.11(95.69 to 98.07) | 75.14(73.53 to 76.70) |
| 20 | 21.29 (20.92 to 24.69) | 96.34 (95.89 to 98.33) | 69.36 (68.34 to 71.70) |
| 21 | 29.93 (28.21 to 31.80) | 99.03 (97.93 to 99.55) | 77.48 (75.97 to 79.04) |
| 22 | 41.09 (36.78 to 45.53) | 96.02 (94.74 to 97.00) | 87.11 (85.83 to 88.31) |
| 23 | 54.91 (50.97 to 58.80) | 98.04 (96.92 to 98.75) | 91.49 (90.42 to 92.48) |
| 24 | 57.27 (53.64 to 60.83) | 98.81 (97.82 to 99.36) | 92.29 (91.26 to 93.23) |
| 25 | 73.33 (68.67 to 77.53) | 97.76 (96.64 to 98.50) | 95.09 (94.24 to 95.85) |
| 26 | 55.21 (51.81 to 58.56) | 99.07 (98.12 to 99.54) | 91.74 (90.68 to 92.72) |
| 27 | 58.28 (54.80 to 61.68) | 99.21 (98.30 to 99.63) | 92.65 (91.64 to 93.57) |
| 28 | 61.41 (57.98 to 64.73) | 99.60 (98.81 to 99.87) | 93.59 (92.64 to 94.46) |
| 29 | 41.23 (38.80 to 43.71) | 99.28 (98.33 to 99.69) | 85.98 (84.67 to 87.23) |
| 30 | 54.64 (51.55 to 57.70) | 99.60 (98.78 to 99.87) | 91.64 (90.58 to 92.62) |
| 31 | 53.48 (50.53 to 56.42) | 99.73 (98.94 to 99.93) | 91.27 (90.19 to 92.27) |
| 32 | 59.48 (56.14 to 62.73) | 99.60 (98.80 to 99.87) | 93.08 (92.10 to 93.98) |
| 33 | 64.92 (61.52 to 68.17) | 100.00 (N/A**)** | 94.60 (93.71 to 95.39) |
| 34 | 62.46 (59.17 to 65.64) | 100.00 (N/A) | 93.99 (93.06 to 94.83) |
| 35 | 70.82 (67.11 to 74.27) | 99.74 (98.99 to 99.93) | 95.75 (94.95 to 96.45) |
| 36 | 61.33 (58.03 to 64.53) | 99.87 (99.08 to 99.98) | 93.65 (92.71 to 94.51) |
| 37 | 67.20(63.65 to 70.55) | 99.87(99.09 to 99.98) | 95.06(94.21 to 95.82) |
| 38 | 68.77(64.83 to 72.45) | 99.10(98.18 to 99.56) | 95.03(94.18 to 95.80) |
| 39 | 42.90(40.59 to 45.25) | 99.71(98.88 to 99.93) | 86.77(85.45 to 87.98) |
| 40 | 60.34(56.09 to 64.45) | 97.94(96.83 to 98.67) | 92.82(91.82 to 93.73) |

Supplementary Table 3. Screening performance of each deep learning model according to prevalence of Italy (6.98%)

| Model No. | PPV, % (95% CI; %) | NPV, % (95% CI; %) | Accuracy, % (95% CI; %) |
| --- | --- | --- | --- |
| 1 | 5.92(2.79 to 12.09) | 92.92(92.55 to 93.29) | 86.08(84.77 to 87.32) |
| 2 | 8.81(6.58 to 11.67) | 93.61(92.72 to 94.41) | 72.69(71.03 to 74.30) |
| 3 | 11.13(10.55 to 11.73) | 99.05(97.79 to 99.59) | 46.94(45.11 to 48.77) |
| 4 | 12.91(11.91 to 13.96) | 98.25(97.07 to 98.96) | 58.23(56.41 to 60.03) |
| 5 | 16.59(15.33 to 17.91) | 98.65(97.67 to 99.22) | 68.32(66.59 to 70.01) |
| 6 | 23.80(21.94 to 25.75) | 98.85(98.02 to 99.34) | 79.52(78.00 to 80.97) |
| 7 | 22.27(20.87 to 23.70) | 99.45(98.72 to 99.76) | 76.58(75.00 to 78.11) |
| 8 | 25.44(23.37 to 27.59) | 98.78(97.95 to 99.28) | 81.32(79.86 to 82.73) |
| 9 | 26.81(24.75 to 28.94) | 98.99(98.20 to 99.43) | 82.27(80.83 to 83.65) |
| 10 | 30.13(26.97 to 33.45) | 97.84(96.96 to 98.48) | 86.15(84.85 to 87.39) |
| 11 | 29.71(27.34 to 32.16) | 98.92(98.14 to 99.38) | 84.64(83.28 to 85.93) |
| 12 | 22.49(20.52 to 24.57) | 98.43(97.53 to 99.01) | 78.78(77.25 to 80.25) |
| 13 | 15.25(13.68 to 16.94) | 97.49(96.39 to 98.26) | 68.60(66.88 to 70.28) |
| 14 | 14.03(12.35 to 15.86) | 96.78(95.64 to 97.64) | 67.93(66.20 to 69.63) |
| 15 | 15.67(13.73 to 17.81) | 96.78(95.70 to 97.59) | 72.27(70.61 to 73.90) |
| 16 | 14.24(13.01 to 15.55) | 98.03(96.91 to 98.75) | 63.78(62.00 to 65.53) |
| 17 | 18.52(16.34 to 20.90) | 97.15(96.14 to 97.91) | 76.39(74.81 to 77.93) |
| 18 | 19.69(18.17 to 21.27) | 98.76(97.86 to 99.28) | 74.13(72.50 to 75.72) |
| 19 | 19.13(17.31 to 21.06) | 98.03(97.05 to 98.69) | 74.98(73.36 to 76.54) |
| 20 | 16.60 (15.16 to 18.13) | 98.21 (97.18 to 98.86) | 69.59 (67.88 to 71.26) |
| 21 | 22.44 (20.97 to 23.95) | 99.34 (98.59 to 99.70) | 77.00 (75.42 to 78.52) |
| 22 | 32.03 (28.21 to 36.08) | 97.28 (96.39 to 97.95) | 87.79 (86.55 to 88.96) |
| 23 | 45.15 (41.24 to 49.08) | 98.66 (97.90 to 99.15) | 91.76 (90.71 to 92.74) |
| 24 | 47.53 (43.86 to 51.19) | 99.20 (98.52 to 99.57) | 92.36 (91.34 to 93.30) |
| 25 | 65.02 (59.68 to 69.97) | 98.47 (97.71 to 98.98) | 95.60 (94.79 to 96.31) |
| 26 | 45.45 (42.07 to 48.83) | 99.37 (98.72 to 99.69) | 91.72 (90.66 to 92.70) |
| 27 | 48.56 (45.02 to 52.08) | 99.46 (98.84 to 99.75) | 92.63 (91.62 to 93.55) |
| 28 | 51.82 (48.24 to 55.34) | 99.73 (99.19 to 99.91) | 93.49 (92.53 to 94.36) |
| 29 | 32.17 (29.98 to 34.40) | 99.51 (98.86 to 99.79) | 85.70 (84.37 to 86.95) |
| 30 | 44.88 (41.81 to 47.94) | 99.73 (99.17 to 99.91) | 91.74 (90.40 to 92.46) |
| 31 | 43.73 (40.82 to 46.64) | 99.82 (99.28 to 99.95) | 91.06 (89.96 to 92.07) |
| 32 | 49.80 (46.36 to 53.20) | 99.73 (99.19 to 99.91) | 92.96 (91.97 to 93.86) |
| 33 | 55.57 (51.92 to 59.13) | 100.00 (N/A) | 94.42 (93.52 to 95.22) |
| 34 | 52.93 (49.46 to 56.33) | 100.00 (N/A) | 93.79 (92.85 to 94.64) |
| 35 | 61.13 (57.95 to 66.10) | 99.83 (99.32 to 99.96) | 95.68 (94.88 to 96.39) |
| 36 | 51.72 (48.29 to 55.13) | 99.91 (99.37 to 99.99) | 93.48 (92.52 to 94.35) |
| 37 | 58.06(54.19 to 61.80) | 99.91(99.39 to 99.99) | 94.93(94.07 to 95.70) |
| 38 | 59.81(55.46 to 63.98) | 99.39(98.77 to 99.70) | 95.13(94.28 to 95.88) |
| 39 | 33.68(31.57 to 35.82) | 99.81(99.24 to 99.95) | 86.40(85.10 to 87.62) |
| 40 | 50.70(46.32 to 55.04) | 98.60(97.83 to 99.10) | 93.17(92.20 to 94.07) |

Supplementary Table 4. Screening performance of each deep learning model according to prevalence of South Korea (0.27%)

| Model No. | | PPV, % (95% CI; %) | NPV, % (95% CI; %) | Accuracy, % (95% CI; %) |
| --- | --- | --- | --- | --- |
| 1 | 0.23(0.10 to 0.49) | | 99.72(99.71 to 99.74) | 91.81(90.76 to 92.78) |
| 2 | 0.36(0.25 to 0.47) | | 99.75(99.72 to 99.79) | 75.68(74.08 to 77.24) |
| 3 | 0.46(0.42 to 0.48) | | 99.96(99.92 to 99.99) | 43.52(41.70 to 45.34) |
| 4 | 0.55(0.49 to 0.58) | | 99.93(99.89 to 99.96) | 56.18(54.35 to 57.99) |
| 5 | 0.73(0.65 to 0.78) | | 99.95(99.91 to 99.97) | 66.91(65.17 to 68.62) |
| 6 | 1.14(1.00 to 1.24) | | 99.96(99.93 to 99.98) | 78.92(77.39 to 80.39) |
| 7 | 1.05(0.94 to 1.11) | | 99.98(99.95 to 99.99) | 75.29(73.68 to 76.85) |
| 8 | 1.25(1.09 to 1.36) | | 99.95(99.92 to 99.97) | 80.94(76.46 to 82.35) |
| 9 | 1.34(1.17 to 1.45) | | 99.96(99.93 to 99.98) | 81.80(80.34 to 83.18) |
| 10 | 1.57(1.31 to 1.78) | | 99.92(99.89 to 99.94) | 87.00(85.72 to 88.20) |
| 11 | 1.54(1.34 to 1.68) | | 99.96(99.93 to 99.98) | 84.41(83.04 to 85.72) |
| 12 | 1.06(0.92 to 1.16) | | 99.94(99.91 to 99.96) | 78.45(76.91 to 79.93) |
| 13 | 0.66(0.57 to 0.73) | | 99.90(99.86 to 99.94) | 68.02(66.29 to 69.71) |
| 14 | 0.60(0.51 to 0.68) | | 99.88(99.84 to 99.91) | 67.79(66.05 to 69.48) |
| 15 | 0.68(0.57 to 0.78) | | 99.88(99.84 to 99.91) | 72.60(70.94 to 74.21) |
| 16 | 0.61(0.54 to 0.66) | | 99.93(99.89 to 99.95) | 62.37(60.58 to 64.13) |
| 17 | 0.83(0.70 to 0.94) | | 99.89(99.86 to 99.92) | 76.85(75.25 to 78.38) |
| 18 | 0.90(0.79 to 0.97) | | 99.95(99.92 to 99.97) | 73.15(71.50 to 74.75) |
| 19 | 0.87(0.75 to 0.95) | | 99.93(99.89 to 99.95) | 74.62(72.99 to 76.19) |
| 20 | | 0.67 (0.64 to 0.79) | 99.91 (99.90 to 99.96) | 68.58 (66.88 to 70.29) |
| 21 | | 1.06 (0.95 to 1.12) | 99.98 (99.95 to 99.99) | 75.77 (74.22 to 77.37) |
| 22 | | 1.71 (1.40 to 2.00) | 99.90 (99.86 to 99.92) | 89.32 (88.14 to 90.42) |
| 23 | | 2.95 (2.47 to 3.36) | 99.95 (99.92 to 99.97) | 92.37 (91.35 to 93.31) |
| 24 | | 3.24 (2.74 to 3.65) | 99.97 (99.95 to 99.98) | 92.53 (91.52 to 93.46) |
| 25 | | 6.43 (5.07 to 7.76) | 99.94 (99.92 to 99.96) | 96.72 (96.01 to 97.34) |
| 26 | | 2.99 (2.55 to 3.33) | 99.98 (99.95 to 99.99) | 91.69 (90.63 to 92.67) |
| 27 | | 3.37 (2.87 to 3.77) | 99.98 (99.96 to 99.99) | 92.58 (91.56 to 93.50) |
| 28 | | 3.83 (3.25 to 4.28) | 99.99 (99.97 to 100.00) | 93.26 (92.29 to 94.14) |
| 29 | | 1.72 (1.52 to 1.86) | 99.98 (99.96 to 99.99) | 85.07 (83.72 to 86.35) |
| 30 | | 2.92 (2.53 to 3.22) | 99.99 (99.97 to 100.00) | 91.10 (90.00 to 92.11) |
| 31 | | 2.79 (2.43 to 3.06) | 99.99 (99.97 to 100.00) | 90.57 (89.45 to 91.61) |
| 32 | | 3.54 (3.02 to 3.94) | 99.99 (99.97 to 100.00) | 92.69 (91.69 to 93.61) |
| 33 | | 4.42 (3.75 to 4.96) | 100.00 (N/A) | 94.01 (93.09 to 94.85) |
| 34 | | 3.99 (3.41 to 4.45) | 100.00 (N/A) | 93.34 (92.37 to 94.22) |
| 35 | | 5.72 (4.74 to 6.57) | 99.99 (99.98 to 100.00) | 95.53 (94.71 to 96.25) |
| 36 | | 3.72 (3.26 to 4.24) | 100.00 (99.98 to 100.00) | 93.09 (92.10 to 93.98) |
| 37 | | 4.87(4.09 to 5.52) | 100.00(99.98 to 100.00) | 94.65(93.77 to 95.44) |
| 38 | | 5.22(4.30 to 6.02) | 99.98(99.96 to 99.99) | 95.34(94.51 to 96.08) |
| 39 | | 1.84(1.64 to 1.97) | 99.99(99.97 to 100.00) | 85.58(84.24 to 86.83) |
| 40 | | 3.663.02 to 4.23) | 99.95(99.92 to 99.97) | 93.96(93.04 to 94.80) |
